# Systematic review and meta-analysis of cognitive assessments used to detect deficits in the bilateral carotid artery stenosis model for vascular cognitive impairment

**DOI:** 10.1177/0271678X251405670

**Published:** 2026-01-17

**Authors:** Matthew J Padgett, Nela Fucelova, Johannes Boltze, Timothy J England, Tuuli Hietamies, Karen Horsburgh, Terence J Quinn, Emily S Sena, Lorraine M Work, Marietta Zille, Rebecca C Trueman, Tracy D Farr

**Affiliations:** 1 School of Life Sciences, University of Nottingham, Nottingham, UK; 2 School of Life Sciences, University of Warwick, Coventry, UK; 3 Stroke, School of Medicine, University of Nottingham, Nottingham, UK; 4 Department of Anaesthesiology, Perioperative, and Pain Medicine, Stanford Medicine, Stanford, CA, USA; 5 Centre for Discovery Brain Sciences, University of Edinburgh, Edinburgh, UK; 6 School of Cardiovascular and Metabolic Health, University of Glasgow, Glasgow, UK; 7 Centre for Clinical Brain Science, University of Edinburgh, Edinburgh, UK; 8 Division of Pharmacology and Toxicology, Department for Pharmaceutical Sciences, University of Vienna, Vienna, Austria; 9 Institute for Neuroscience and Cardiovascular Research, Queen’s Medical Research Institute, University of Edinburgh, Edinburgh, UK

**Keywords:** Behaviour, bilateral carotid artery stenosis, cognition, meta-analysis, vascular dementia

## Abstract

Hypoperfusion via bilateral carotid artery stenosis is the most common mouse model of vascular cognitive impairment, but the literature varies surrounding which behavioural tests are most appropriate to detect cognitive deficits in this model. We aimed to address this via a systematic review and meta-analysis. We also aimed to provide a recommendation that also considers how the tests cover the different cognitive domains. We identified 1714 publications and extracted data from 56. Interestingly, only six cognitive behavioural tests were employed across the literature with the most common being the Morris water and radial arm mazes, followed by the *Y* maze, novel object recognition, open field, and the Barnes maze. While all examined tests were able to detect cognitive impairments in hypoperfused mice, there was a high degree of heterogeneity across the publications, highlighting that not all research groups consistently observed cognitive deficits in the model. There was also evidence of publication bias, and occasionally some publications with extremely high effect sizes were influential. We recommend all tests, but ideally experiments should be complemented with additional approaches that examine a greater range of cognitive functions.

## Introduction

Animal models have contributed to our understanding of the pathophysiology of vascular cognitive impairment (VCI).^1,2^ The most common rodent models are based around hypoperfusion, as this is presumed to be a key driver of VCI. The validity of hypoperfusion models is supported as they feature some pathological changes associated with VCI, such as white matter damage and cognitive deficits.^3^ However, there is only incomplete knowledge regarding which behavioural tests are best suited to investigate impact and outcome in experimental studies on VCI, and a pressing need to identify behavioural test batteries that are universally interpreted and reported.^4,5^ There is a large variety of cognitive behavioural tests available with different implementation practices and multiple outcome measures. Furthermore, the choice of outcome assessments in rodents may include pragmatic considerations rather than preferences surrounding the cognitive domains covered by the tests, or their clinical relevance.

We previously undertook a systematic review and reported that approximately 20 different behavioural tests were employed across preclinical VCI research.^6^ The most common test was the Morris water maze (MWM) followed by the novel object recognition (NOR) test and *T*, *Y* or radial arm mazes (RAM); some papers also employed sensorimotor assessments. Interestingly, this previous work also suggested that mouse models of VCI were more widely used than rats. Since then, there has been an increasing number of publications that have employed the mouse bilateral carotid artery stenosis (BCAS) model.^7^ However, behavioural testing in mice is not without challenges. To date, cognitive behavioural testing outcome reports in the BCAS model have been variable. The earliest report that comprehensively characterised behaviours in BCAS mice reported no deficits in sensorimotor outcomes (hot plate, rotarod, grip strength), locomotion (open field), aspects of memory associated with fear (light/dark transition, startle response and fear conditioning), or learned helplessness (Porsolt).^8^ However, BCAS mice made significantly more revisiting errors in the RAM. The RAM examines spatial learning and memory as well as working memory as it requires mice to obtain food rewards from the end of arms in a star shaped maze without returning to locations from which rewards have already been collected.^9^ Other studies have since reported RAM deficits in BCAS mice,^10–12^ though there are occasional reports that do not detect impairments.^13,14^ Spatial learning and memory is traditionally explored using the MWM.^15^ The classic approach (place learning) requires rodents to find a submerged or hidden escape platform with a fixed location across several days. Several publications suggest the MWM is effective at detecting spatial memory impairments with place learning.^16–18^ However, there are also reports in which BCAS mice did not exhibit deficits in spatial learning, reference memory or serial learning of novel platform locations in the MWM.^10^ We have also failed to detect spatial memory deficits in the MWM^19^ unless a more severe (160 μm compared to the standard 180 μm diameter microcoils)^20^ or prolonged period of hypoperfusion (6 months compared to the typical 1 m endpoint) was employed that also resulted in the presence of subcortical vascular lesions and atrophy^21^; this aligns with another report.^22^

Being confident in the ability of the different cognitive behavioural tests to detect impairments in the BCAS model is essential to allow researchers to make informed decisions surrounding outcome measures in preclinical VCI research, and to appropriately relate outcomes to cognitive domains. We performed a systematic review and meta-analysis to determine which cognitive behavioural tests are most used in the BCAS model, and which were most reliable at identifying impairments via the estimate of effect size and variance of the outcome measures. We assessed publication bias and analysed the impact of several covariates on the results in order to make recommendations and consider how the tests cover different cognitive domains.

## Material and methods

The protocol was registered with PROSPERO (CRD420222422030), followed recommended procedures,^23^ and conformed to the PRISMA reporting guidelines (Supplemental Tables 1 and 2). The literature search, screening and data extraction were all conducted by two independent authors (MJP, NF) and discrepancies were managed by a third author (TDF).

### Search strategy

The search was performed on 5 February 2021 using the search engine PubMed and the online databases MEDLINE (Ovid), Web of Science (Clarivate) and Scopus (Elsevier) without any limits on publication dates. The following search terms were used: (Bilateral carotid artery stenosis OR BCAS OR chronic cerebral hypoperfusion OR chronic hypoperfusion OR cerebral hypoperfusion) AND (Vascular cognitive impairment OR VCI OR Vascular dementia OR VaD OR small vessel disease OR SVD).

During Phase 1, duplicate papers were removed alongside any not available in English. The titles and abstracts were screened for the following inclusion criteria: use of mice (male or female and above 4 weeks of age), permanent BCAS without other interventions/treatments, an appropriate control group for comparison (untreated sham, naïve or baseline), and behavioural tests that assess any aspect of cognition. The following exclusion criteria were applied: no original data, human studies, use of rats or species other than mice and use of alternative stenosis or dementia models. When this could not be determined from the title and abstract, the publications automatically entered Phase 2, which involved a full text screening for the same criteria.

### Quality score

Included publications were given a quality score using a modified version of the Collaborative Approach to Meta Analysis and Review of Animal Experimental Studies (CAMARADES) quality score for preclinical studies in stroke research.^24^

1. Publication in a peer-reviewed journal.
2. Randomisation of BCAS or sham and/or treatment and control.
3. Blinded assessment of behavioural outcome.
4. A priori sample-size calculations.
5. Statement of compliance with regulatory requirements/animal welfare law compliance.
6. Statement regarding possible conflict of interest.

One point was allocated for each reported item for a maximum possible score of 6, and quality score was used as a co-variate in a meta-regression.

### Data extraction

Extracted study characteristics included: Study ID (first author, year of publication, journal and DOI), subjects (species, strain, sex, age and/or weight), as well as size of microcoil used to produce stenosis (0.16, 0.17, 0.18, 0.20, 0.5 mm).

The primary outcome measures were performance in the cognitive behavioural tests and the timepoints they were acquired in relation to the BCAS procedure. Secondary measures included which cognitive behavioural tests were used.

For behavioural tests reported in more than 10 studies, the mean performance of each outcome measure was extracted alongside the unit of measure, standard error or deviation and sample size. When these values were not directly reported, screenshots of the Figures were taken, and data was extracted manually using ImageJ software (NIH, USA). If this data could not be extracted, the authors were contacted. Data could not be extracted from one publication as there was no record of sample size, and we were unable to reach the authors.

### Data analysis

All outcome measures were examined to ensure there was <10% absolute difference between two independent authors. When this was confirmed, both measures were averaged. If there was >10% absolute difference, a third reviewer extracted the data, and this value was used.

All analyses were performed using the metafor Meta-Analysis Package for R (v2.4-0). Hedges G and sample variance were calculated for all outcome measures and when deficits were indicated by higher scores, Hedges G was inverted by multiplying by −1. Multiple measures for the same outcome within the same behavioural test were combined using a nested approach to obtain a fixed effects size for each test in each study. Subsequently, the nested fixed effects were used in a random effects model and heterogeneity was expressed as the *I*^2^ statistic.

Publication bias was examined with funnel plots and asymmetry was assessed via Egger’s regression. Funnel plots were supplemented by trim-and-fill analysis to estimate studies that may have been excluded from the literature. The leave-one-out analysis was also performed to highlight influential studies by iteratively re-fitting the random effects model when each individual study was excluded.

There are many potential factors that could impact study outcome. We identified the following key covariates: research group, species, strain, age, sex, microcoil size, comorbidities, quality scores, randomisation, blinding and the timepoint at which the behavioural assessment was conducted. We proposed to use these covariates in a mixed-effects meta-regression to formally evaluate the degree to which they predict heterogeneity within the model and whether the prediction is significant. Retrospectively, several covariates were excluded as there was insufficient variability in these measures across included papers (research group, strain, age, sex, comorbidities and several aspects of the quality score). Ultimately, microcoil size, timepoint, blinding, randomisation and sample size calculations from the quality score were included as moderators in the meta-regression. Sample size calculations and microcoil size were unfortunately excluded as moderators for the MMW and RAM, respectively, due to the lack of variability across included studies. Prior to the meta-regression, the covariates were assessed for collinearity by running a pair-wise correlation matrix; none of the covariates were correlated.

## Results

### The publications using the BCAS model employed relatively few behavioural tests and had similar study characteristics

**Figure 1.**
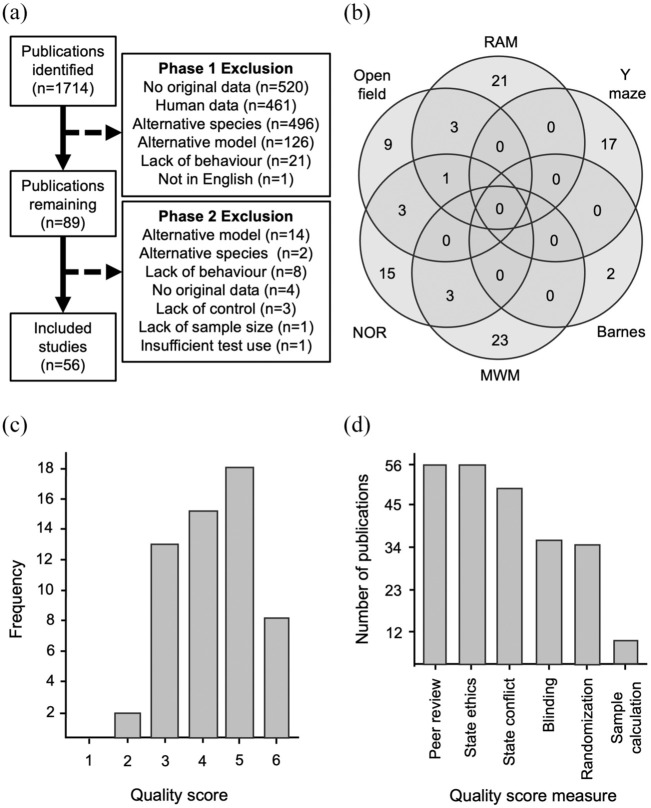
Study search and characteristics. (a) Flow diagram depicting study exclusions. (b) Venn diagram depicting the usage of the different behavioural tests across the studies. (c) Frequency of the number of studies that achieved the different possible quality scores. (d) Number of studies that achieved each individual quality score measure.

Ultimately, 56 publications underwent data extraction (Figure 1(a), Supplemental Table 3). There were six different cognitive behavioural tests employed across all studies. Interestingly, papers rarely used combinations of these tests (Figure 1(b)). The most widely employed tests were the MWM (23/56, of which 16 included data for the probe trial) and the RAM (21/56). The Y maze (17/56) and NOR (15/56) were also used often, and to a lesser extent, open field (9/56) and the Barnes maze (2/56).

The median quality score for all publications was 4 and the mode was 5. Only 8/56 papers scored the maximum of 6 (Figure 1(c)). All studies were published in a peer-reviewed journal and mentioned compliance with ethical regulations, and most (50/56) provided a statement regarding conflict of interest (Figure 1(d)). Blinding (36/56) and randomisation (35/56) were reasonably well reported, but power calculations to estimate sample size (8/56) were rare (Figure 1(d)).

### All behavioural tests detected cognitive deficits in the BCAS model, though there was high heterogeneity across publications

**Figure 2.**
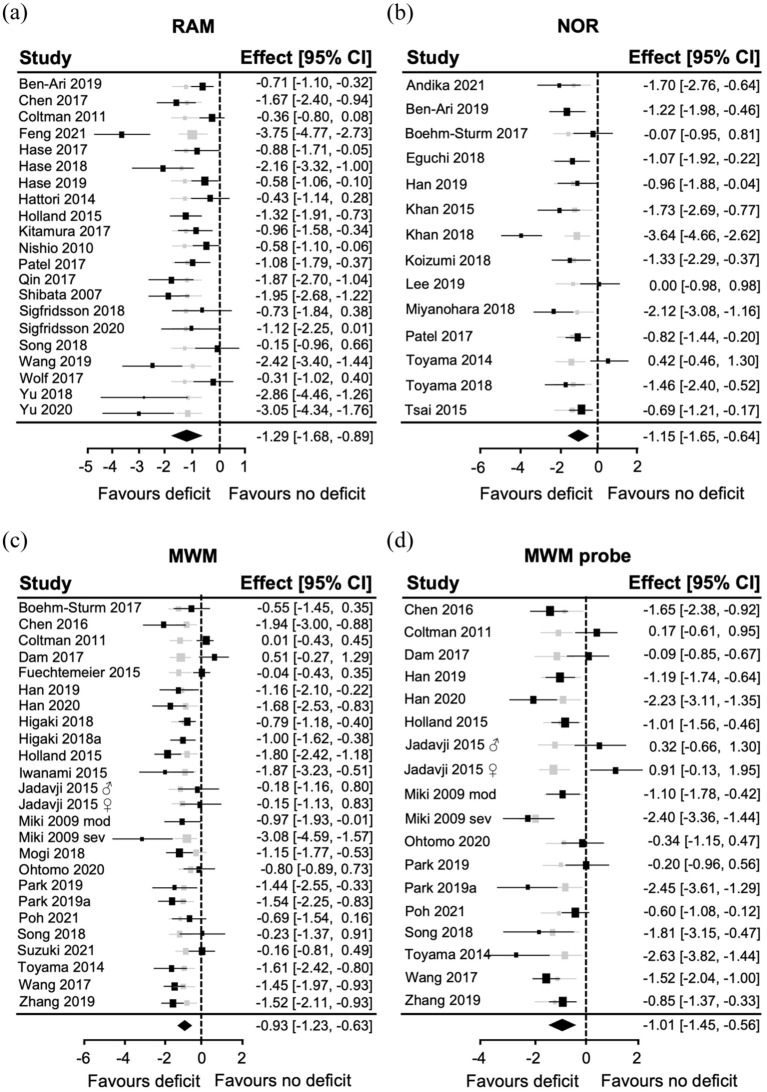
Summary of the utility of the RAM, NOR and MWM to detect deficits in BCAS mice. Forest plots for (a) RAM, (b) NOR, (c) MWM and (d) MWM probe trial displaying individual study effect sizes (squares) with 95% confidence interval (CI). The pooled overall effect size is indicated by the diamond. Gray squares indicate adjusted overall effect size when that study is omitted (leave-one-out analysis).

The RAM exhibited a large overall effect size −1.29 (95% CI: −1.68, −0.89; Figure 2(a)). All individual studies observed RAM deficits in the BCAS group. There were a cluster of five studies that exhibited very large effect sizes (between 2 and 4), but another five that were very low. Indeed, there was high heterogeneity across the papers (*I*^2^ = 84.53%), and the Leave-one-out analysis suggested that extreme studies impacted the overall effect size (Figure 2(a)). Another large effect size was observed with NOR −1.15 (95% CI: −1.65, −0.64; Figure 2(b)). There were three individual studies with effect sizes greater than two that were influential, but as two studies did not observe a deficit, heterogeneity remained high (*I*^2^ = 80.35%). The smallest effect size of any of the cognitive tests was observed for the MWM −0.93 (95% CI: −1.23, −0.63; Figure 2(c)). There were three studies with large effect sizes, two that reported no deficits in BCAS mice, and six that were very near 0. This contributed again to high heterogeneity (*I*^2^ = 76.76%). The MWM probe trial was also effective at identifying deficits in the BCAS group −1.01 (95% CI: −1.45, −0.56; Figure 2(d)). There was a high heterogeneity (*I*^2^ = 84.79%) as four influential studies had large, three had small effect sizes, and another three did not identify deficits in the BCAS group.

**Figure 3.**
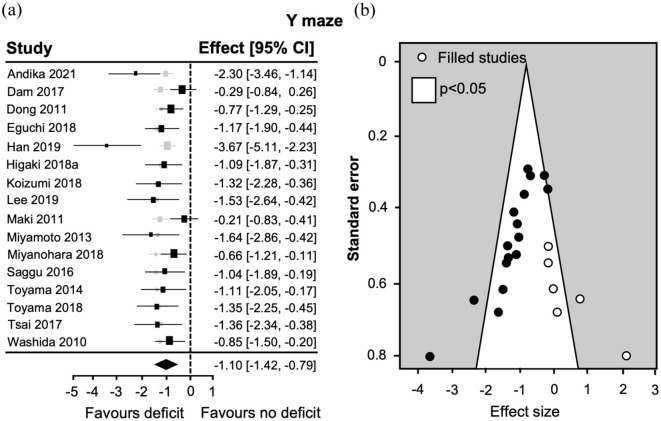
Summary of the utility of the *Y* maze to detect deficits in BCAS mice and potential publication bias. (a) Forest plot displaying individual study effect sizes (squares) with 95% confidence interval (CI). The pooled overall effect size is indicated by the diamond. (b) Funnel plot expressing standard error as a function of the effect size for individual studies (circles). Gray squares indicate adjusted overall effect size when that study is omitted (leave-one-out analysis), and open circles in the funnel plot represent publications added by trim-and-fill analysis.

The overall effect size for the *Y* maze was also large −1.10 (95% CI: −1.42, −0.79; Figure 3(a)), but heterogeneity was lower (*I*^2^ = 59.01%) suggesting slightly more reproducible findings across studies.

### Publication bias was evident for some of the cognitive behavioural tests in the literature

Despite the outcomes being slightly more reproducible in studies using the *Y* maze, funnel plot asymmetry was observed (Figure 3(b)), suggesting publication bias. Indeed, the Egger’s regression was significant (*z* = −5.21, *p* < 0.0001) and six theoretical studies were added by a trim-and-fill analysis (Figure 3(b)). Interestingly, the leave-one-out analysis showed that studies with the highest effect sizes were not influential (Figure 3(a)).

**Figure 4.**
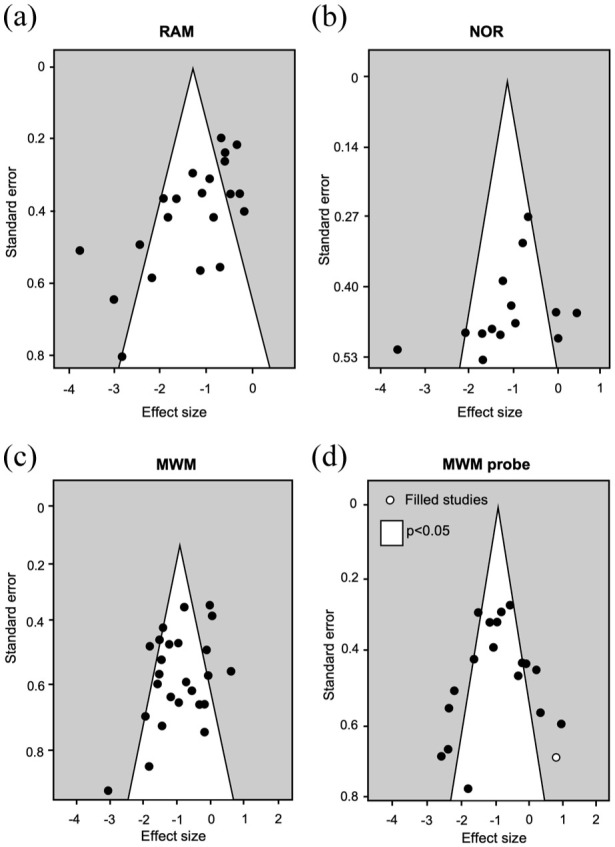
Publication bias in the RAM, NOR and MWM. Funnel plots expressing standard error as a function of the effect size for individual studies (circles) for the (a) RAM, (b) NOR, (c) MWM and (d) MWM probe trial. Open circles in the funnel plot represent publications added by trim-and-fill analysis.

Potential publication bias was visible for the RAM studies via funnel plot asymmetry (Figure 4(a)) and the resulting Egger’s regression determined this was significant (*z* = −3.84, *p* < 0.0001). The leave-one-out analysis also suggested that some individual studies with the highest effect sizes were influential (Figure 2(a)). Some funnel plot asymmetry was observed for the NOR test, but Egger’s regression was not significant (*z* = −1.37, *p* = 0.17; Figure 4(b)). The leave-one-out analysis was balanced as none of the studies were particularly influential in driving the effect size (Figure 2(b)).

Mild funnel plot asymmetry was observed for the MWM and MWM probe trial (Figure 4(c) and (d)), but neither Egger’s regression was significant: (*z* = −1.69, *p* = 0.14) and (*z* = −0.1, *p* = 0.32, respectively). This suggests minimal publication bias, though one theoretical study without a BCAS deficit was added with trim-and-fill to the MWM probe plot (Figure 4(d)). The leave-one-out analysis revealed influential studies both in favour of BCAS deficits and in favour of no deficits for the MWM probe trial but none for the MWM (Figure 2(c) and (d)).

### Covariates impacted the analysis for several behavioural tests

**Figure 5.**
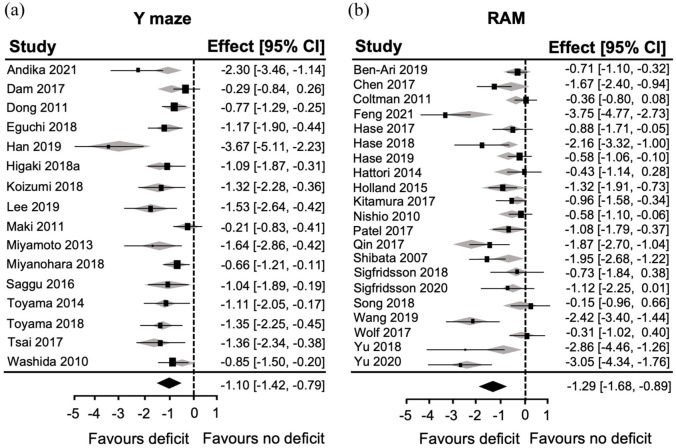
Summary of the contribution of moderators on the effect size. Forest plots for the (a) *Y* maze and (b) RAM displaying individual study effect sizes (squares) with 95% confidence interval (CI). The pooled overall effect size is indicated by the diamond. Gray diamonds indicate adjusted overall effect size when moderators were taken into account.

The meta-regression suggested that the moderators explained 0, 29, and 28% of the heterogeneity for the MWM, MWM probe trial and NOR, respectively, but none achieved significance. Interestingly, 75% of the heterogeneity in the *Y* maze data was explained by the moderators, and this was significant (QM(6)18.99, *p* = 0.0042; Figure 5(a)). The final timepoint at which the behavioural test was conducted (also the duration of the hypoperfusion procedure) had the largest impact; effect size reduced with time following BCAS surgery (Table 1). This was closely followed by microcoil size (Table 1) and a 0.17 mm diameter microcoil produced greater effect sizes than 0.16 and 0.18 mm. Further to this, 59% of the heterogeneity was predicted by the moderators in the RAM (QM(4)20.89, *p* = 0.0003; Figure 5(b)), but only sample size calculations remained significant. Reporting sample size calculations was associated with lower effect sizes (Table 1). Indeed, the studies with some of the highest effect sizes did not perform sample size calculations.

**Table 1.** Effect size for the *Y* maze and the RAM when moderators were accounted for. The coefficient represents the estimated change in effect size associated with a one-unit change in the moderator.

Behavioural test	Moderator	Coefficient (95% CI)	Standard error	*p*-Value
*Y* maze	Timepoint	−0.0792 (−0.1205, −0.0379)	0.0211	0.0002
	Blinding	−0.6132 (−1.3454, 0.1191)	0.3736	0.1008
	Randomisation	0.1881 (−0.5349, 0.9111)	0.3689	0.6101
	Sample size	−0.7311 (−1.6287, 0.1664)	0.4580	0.1104
	Microcoil size (0.17)	2.2789 (0.7130, 3.8447)	0.7989	0.0043
	Microcoil size (0.18)	1.4670 (0.5702, 2.3638)	0.4576	0.0013
RAM	Timepoint	−0.0035 (−0.0111, 0.0042)	0.0039	0.3718
	Blinding	−0.1040 (−0.9145, 0.7065)	0.4135	0.8014
	Randomisation	0.5004 (−0.3314, 1.3322)	0.4244	0.2384
	Sample size	−1.9749 (−2.8690, −1.0809)	0.4561	0.0001

## Discussion

This systematic review and meta-analysis aimed to identify which behavioural tests were most widely used and effective at detecting cognitive impairments in the BCAS model. We identified and extracted data from 56 publications. Interestingly, the literature exhibited little diversity in the behavioural tests employed considering the wide range of rodent cognitive assessment methods that are available. The RAM, MWM, NOR, Y maze, open field and Barnes maze were employed, though the two latter were not reported in a sufficient number of papers to analyse. All four behavioural tests were effective at detecting deficits in BCAS mice, though there was a high degree of heterogeneity across studies and some evidence of publication bias as well as the presence of influential studies.

Our analysis found the RAM to be the most widely used behavioural test, and it was very effective at detecting cognitive deficits in the BCAS model. There was also a high level of heterogeneity among publications that reported RAM data with some exhibiting very large^12,25,26^ and others small effect sizes.^10,13,14,27^ Indeed, the leave-one-out analysis revealed some of the studies with the largest effect sizes were influential. There was further evidence of publication bias, which is well known to overstate efficacy.^28^ The prevalence of RAM across the literature may be due to the earliest report in which the RAM emerged as the only test from a large battery was able to identify BCAS deficits.^8^ This landmark paper also proposed that since no hippocampal damage was observed, the working memory deficits must be due to hypoperfusion induced damage to the white matter and frontal-subcortical circuits. This is also well aligned with the view that people living with VCI are impacted by impairments in the Executive Function Cognitive Domain. However, the RAM is also sensitive to spatial learning and memory, and the hippocampus is well known to be vulnerable to hypoperfusion via the wide body of literature on global cerebral ischemia, reviewed elsewhere.^29^ This seems to have been considered across the literature as the classic place learning paradigm in the MWM was the second most commonly employed behavioural test. Interestingly, despite the overall ability of the MWM to detect spatial memory deficits in the Learning and Memory Cognitive Domain, there was still high heterogeneity, and several papers did not observe deficits in BCAS mice.^13,19,20,30,31^ The heterogeneity likely contributed to the insignificant publication bias. Of note, some groups that did not report deficits^10,19,31,32^ were subsequently able to detect them when the severity^20^ or duration^21,22^ of hypoperfusion was increased. Interestingly, two^22,33^ of three^16^ publications that employed more severe hypoperfusion also reported deficits in the MWM using a cued, visible platform task. Though the animals were still able to learn, there is the possibility that visual impairments contributed to the reported cognitive deficits. Indeed, retinal damage has been reported in this model.^34,35^ Finally, some publications also employed other MWM paradigms that begin to incorporate aspects of working memory.^10,36^ Serial learning (match to place or sample) involves moving the platform location daily and training to this new criterion. In both publications, BCAS mice did not exhibit any deficits with learning the changing platform location, suggesting the MWM may not be ideal to assess working memory in this model.

The MWM probe trial also falls into the Learning and Memory Cognitive Domain, but is particularly sensitive to spatial reference or semantic memory (also generally attributed to the hippocampus) as it involves removal of the platform at the end of the test period. The probe trial exhibited a slightly larger effect size than the classic MWM, it was also associated with higher heterogeneity and there were a greater number of influential studies. The reason for this is not clear, but it could be impacted by sample size. Despite the additional information the probe trial can provide, it was not performed in all studies that used the MWM. There was also a wide variety of different possible outcome measures reported for the probe trial. These included time in target quadrant, latency to enter the target quadrant, and various methods to assess the number of platform crossings. While we nested to account for multiple outcomes in the meta-analysis, we cannot exclude the possibility that this could have offered more opportunities to detect differences from the same data. Due to the aversive nature of the MWM, an alternative dry approach such as the Barnes maze may be ideal. However, we only identified two publications that employed this test, which was insufficient to extract data or conduct a meta-analysis.

The NOR task is sensitive to recognition memory.^37^ It requires animals to spontaneously explore two identical objects and after a reasonably short period of time, one of the objects is replaced.^38^ Rodents tend to explore the novel object to a greater degree. There was a large overall effect size with NOR, some heterogeneity, and no publication bias. Despite assessing a different type of memory, this test still falls into the Learning and Memory Cognitive Domain. One feature of this test that could make it more desirable is the ability to incorporate social odour cues as a motivator thus allowing it to cover the Social Cognition and Emotion Cognitive Domain.^39^ There was only one publication that used this version of the task,^20^ albeit there was a very small effect size.

The *Y* maze relies on spontaneous exploration of three arms^40^ and mice should exhibit the tendency to alternate arm choices.^41^ There is a spatial learning and memory component that covers the Learning and Memory Cognitive Domain as well as aspects of working memory (Executive Function Cognitive Domain). There was a large overall effect size in the *Y* maze, but a much lower heterogeneity as most reports were consistent. Unsurprisingly, there was also a significant publication bias, and six potential studies were added with trim-and-fill. Overall, this suggests literature employing the *Y* maze is reproducible, but prone to publication bias. The open field test was unfortunately only used by nine different studies and thus was not included in the meta-analysis. This is disappointing because it is sensitive to anxiety and could also cover the Social Cognition and Emotion Cognitive Domain. However, most of the outcome measures tended to focus on total distance travelled, which is more indicative of locomotor ability. Three papers measured speed,^14,30,42^ and BCAS mice tended to be faster than sham controls. This may be due to increased anxiety or hyperexcitability. A few groups did look at the amount of time spent in the edges of the arena, as this thigmotactic behaviour has been proposed as a measure of anxiety.^43^ However, the results were not consistent as two publications reported no differences in centre/edge seeking behaviour between BCAS and sham mice,^8,31^ whereas the other two reported that BCAS mice spent more time near the edges.^42,44^

There are limitations to the present study. While we identified a reasonable number of studies overall, there were not large numbers of studies that used each individual behavioural test. This could be perceived as a limitation. However, the heterogeneity was high, which could equally suggest our sample represents the literature well. One strong limitation is that we made some changes to the initial proposal that was published in PROSPERO. These changes were not pre-planned and could be introducing bias. However, we deemed them necessary as they surround essential considerations across the body of work we are examining. There are many factors that could affect study outcome. These can be at the level of subject such as strain, age, sex or comorbidities, as well as procedural differences such as microcoil size employed, the duration of the hypoperfusion, or even variations in how behavioural testing was performed. Experimental design can also impact outcome, for example, consideration towards measures to reduce bias. Our initial PROSPERO proposal considered an extensive list of modifying factors, and the aim was to use meta-regression to determine which ones significantly predicted heterogeneity. Retrospectively, they were far too numerous to be realistically employed. But more importantly, several covariates needed to be excluded as there was limited variation across the included studies. For example, almost all the studies used mice with a *C57BL6* background in a narrow age range (under 12 weeks). Only three studies employed female animals,^14,31,32^ and from these, only one^31^ separated them for analysis purposes. Other covariates were excluded as they were difficult to quantify. From the 56 included papers, we identified approximately 28 different research groups. However, this was subjective, as it was taken from last author affiliations and several collaborators/affiliations were listed for each publication, alongside movement of some authors across organisations. As such, we refined our list of potential covariates to microcoil size and timepoint at which the behavioural tests were conducted (generally also the duration of the hypoperfusion). We also broke down quality score and used blinding, randomisation, and sample size calculations as moderators. Despite the best of intentions, the meta-regression still needs to be interpreted with caution. For example, the moderators only significantly predicted heterogeneity for the RAM and *Y* maze. Furthermore, the breakdown of individual moderators is not always easy to explain. While sample size calculations to properly power a study were associated with lower effect sizes in publications employing RAM, effect sizes were reduced with longer durations of hypoperfusion in the *Y* maze. This is likely due to the low number of studies with significant variation in the moderators. Other limitations surround factors that were unable to consider. There are a wide variety of procedural differences with regards to conducting behavioural tests. Unfortunately, these were not always consistently reported across the publications, but even if we had tried to incorporate this, the results of the meta-regression already suggest there are too few studies to appropriately draw meaningful conclusions. We also did not design our meta-analysis to consider histological outcomes and this means we can only make conclusions surrounding the behavioural tests without consideration to the model in it’s entirety.

In conclusion, all four behavioural tests were able to detect deficits in BCAS mice, but it remains challenging to confidently recommend any single test (Supplemental Table 4 summarises our findings and reccomendations). Publication bias was evident for the RAM, MWM and in particular for the *Y* maze. While this may have exaggerated effect sizes, there was still high heterogeneity among publications and deficits in BCAS mice were not consistent. All the tests assess some form of memory, which means the Learning and Memory Cognitive Domain is well represented. However, this is heavily biased towards spatial learning and memory. Only the RAM, Y maze and modified MWM are able to incorporate working memory aspects of the Executive Function Cognitive Domain. Incorporating additional tests that better assess other aspects of the Executive Function Cognitive Domain, like cognitive flexibility, would better reflect the breadth of deficits experienced by people living with dementia. One way to do this is to use operant testing boxes or touch screen boxes, in which protocols can be developed that are similar to the human Cambridge Neuropsychological Test Automated Battery (CANTAB).^45,46^ Finally, practical aspects can also affect the decision to use a behavioural test. For example, the RAM, *Y* maze and MWM require specialist equipment, and the RAM requires extensive training regimes and food restriction to motivate performance. This may make it less desirable for widespread implementation. It should also be noted that all tests require the visual system. Assessment of visual function needs to be included in test batteries, and if deficits are found, the tasks should be adapted. Our final recommendation would be to include a battery of cognitive tests that examine a combination of cognitive domains, but it may not be possible to consistently identify deficits.

## Supplemental Material

sj-docx-1-jcb-10.1177_0271678X251405670 – Supplemental material for Systematic review and meta-analysis of cognitive assessments used to detect deficits in the bilateral carotid artery stenosis model for vascular cognitive impairment Supplemental material, sj-docx-1-jcb-10.1177_0271678X251405670 for Systematic review and meta-analysis of cognitive assessments used to detect deficits in the bilateral carotid artery stenosis model for vascular cognitive impairment by Matthew J Padgett, Nela Fucelova, Johannes Boltze, Timothy J England, Tuuli Hietamies, Karen Horsburgh, Terence J Quinn, Emily S Sena, Lorraine M Work, Marietta Zille, Rebecca C Trueman and Tracy D Farr in Journal of Cerebral Blood Flow & Metabolism
