# Supplementary material for: Systematic review and meta-analysis of cognitive assessments used to detect deficits in the bilateral carotid artery stenosis model for vascular cognitive impairment
Source: J Cereb Blood Flow Metab. 2026 Jan 17:0271678X251405670. Online ahead of print. doi: 10.1177/0271678X251405670 (PMC12812064; doi:10.1177/0271678X251405670)
Supplement: sj-docx-1-jcb-10.1177_0271678X251405670 – Supplemental material for Systematic review and meta-analysis of cognitive assessments used to detect deficits in the bilateral carotid artery stenosis model for vascular cognitive impairment [file sj-docx-1-jcb-10.1177_0271678X251405670.docx]

**Supplementary Table 1.** PRISMA Abstract checklist.


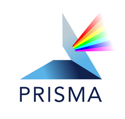


**PRISMA 2020 Abstract Checklist**

| **Section and Topic** | **Item #** | **Checklist item** | **Reported (Yes/No)** |
| --- | --- | --- | --- |
| **TITLE** | | |  |
| Title | 1 | Identify the report as a systematic review. | Yes |
| **BACKGROUND** | | |  |
| Objectives | 2 | Provide an explicit statement of the main objective(s) or question(s) the review addresses. | Yes |
| **METHODS** | | |  |
| Eligibility criteria | 3 | Specify the inclusion and exclusion criteria for the review. | No |
| Information sources | 4 | Specify the information sources (e.g. databases, registers) used to identify studies and the date when each was last searched. | No |
| Risk of bias | 5 | Specify the methods used to assess risk of bias in the included studies. | No |
| Synthesis of results | 6 | Specify the methods used to present and synthesise results. | No |
| **RESULTS** | | |  |
| Included studies | 7 | Give the total number of included studies and participants and summarise relevant characteristics of studies. | Yes |
| Synthesis of results | 8 | Present results for main outcomes, preferably indicating the number of included studies and participants for each. If meta-analysis was done, report the summary estimate and confidence/credible interval. If comparing groups, indicate the direction of the effect (i.e. which group is favoured). | Yes |
| **DISCUSSION** | | |  |
| Limitations of evidence | 9 | Provide a brief summary of the limitations of the evidence included in the review (e.g. study risk of bias, inconsistency and imprecision). | No |
| Interpretation | 10 | Provide a general interpretation of the results and important implications. | Yes |
| **OTHER** | | |  |
| Funding | 11 | Specify the primary source of funding for the review. | No |
| Registration | 12 | Provide the register name and registration number. | No |

*From:*  Page MJ, McKenzie JE, Bossuyt PM, Boutron I, Hoffmann TC, Mulrow CD, et al. The PRISMA 2020 statement: an updated guideline for reporting systematic reviews. BMJ 2021;372:n71. doi: 10.1136/bmj.n71. This work is licensed under CC BY 4.0. To view a copy of this license, visit <https://creativecommons.org/licenses/by/4.0/>

**Supplementary Table 2.** PRISMA Checklist.

| **Section and Topic** | **Item #** | **Checklist item** | **Location where item is reported** |
| --- | --- | --- | --- |
| **TITLE** | | |  |
| Title | 1 | Identify the report as a systematic review. | Page 1 |
| **ABSTRACT** | | |  |
| Abstract | 2 | See the PRISMA 2020 for Abstracts checklist. | Supp Table 2 |
| **INTRODUCTION** | | |  |
| Rationale | 3 | Describe the rationale for the review in the context of existing knowledge. | Page 3 |
| Objectives | 4 | Provide an explicit statement of the objective(s) or question(s) the review addresses. | Pages 5-6 |
| **METHODS** | | |  |
| Eligibility criteria | 5 | Specify the inclusion and exclusion criteria for the review and how studies were grouped for the syntheses. | Page 8 |
| Information sources | 6 | Specify all databases, registers, websites, organisations, reference lists and other sources searched or consulted to identify studies. Specify the date when each source was last searched or consulted. | Page 7 |
| Search strategy | 7 | Present the full search strategies for all databases, registers and websites, including any filters and limits used. | Page 7 |
| Selection process | 8 | Specify the methods used to decide whether a study met the inclusion criteria of the review, including how many reviewers screened each record and each report retrieved, whether they worked independently, and if applicable, details of automation tools used in the process. | Page 7 |
| Data collection process | 9 | Specify the methods used to collect data from reports, including how many reviewers collected data from each report, whether they worked independently, any processes for obtaining or confirming data from study investigators, and if applicable, details of automation tools used in the process. | Pages 7 and 10 |
| Data items | 10a | List and define all outcomes for which data were sought. Specify whether all results that were compatible with each outcome domain in each study were sought (e.g. for all measures, time points, analyses), and if not, the methods used to decide which results to collect. | Page 9-11 |
|  | 10b | List and define all other variables for which data were sought (e.g. participant and intervention characteristics, funding sources). Describe any assumptions made about any missing or unclear information. | Page 9 |
| Study risk of bias assessment | 11 | Specify the methods used to assess risk of bias in the included studies, including details of the tool(s) used, how many reviewers assessed each study and whether they worked independently, and if applicable, details of automation tools used in the process. | Pages 8-9 |
| Effect measures | 12 | Specify for each outcome the effect measure(s) (e.g. risk ratio, mean difference) used in the synthesis or presentation of results. | Page 10 |
| Synthesis methods | 13a | Describe the processes used to decide which studies were eligible for each synthesis (e.g. tabulating the study intervention characteristics and comparing against the planned groups for each synthesis (item #5)). | Page 8 |
|  | 13b | Describe any methods required to prepare the data for presentation or synthesis, such as handling of missing summary statistics, or data conversions. | Page 10 |
|  | 13c | Describe any methods used to tabulate or visually display results of individual studies and syntheses. | Page 10 |
|  | 13d | Describe any methods used to synthesize results and provide a rationale for the choice(s). If meta-analysis was performed, describe the model(s), method(s) to identify the presence and extent of statistical heterogeneity, and software package(s) used. | Page 10-11 |
|  | 13e | Describe any methods used to explore possible causes of heterogeneity among study results (e.g. subgroup analysis, meta-regression). | Page 11 |
|  | 13f | Describe any sensitivity analyses conducted to assess robustness of the synthesized results. | Pages 11-12 |
| Reporting bias assessment | 14 | Describe any methods used to assess risk of bias due to missing results in a synthesis (arising from reporting biases). | Page 11 |
| Certainty assessment | 15 | Describe any methods used to assess certainty (or confidence) in the body of evidence for an outcome. | Pages 11-12 |

| **RESULTS** | | |  |
| --- | --- | --- | --- |
| Study selection | 16a | Describe the results of the search and selection process, from the number of records identified in the search to the number of studies included in the review, ideally using a flow diagram. | Page 13 & Figure 1 |
|  | 16b | Cite studies that might appear to meet the inclusion criteria, but which were excluded, and explain why they were excluded. | Page 13 & Figure 1 |
| Study characteristics | 17 | Cite each included study and present its characteristics. | Supp Table 3 |
| Risk of bias in studies | 18 | Present assessments of risk of bias for each included study. | Supp Table 3 |
| Results of individual studies | 19 | For all outcomes, present, for each study: (a) summary statistics for each group (where appropriate) and (b) an effect estimate and its precision (e.g. confidence/credible interval), ideally using structured tables or plots. | Figures 2, 3, & 5 |
| Results of syntheses | 20a | For each synthesis, briefly summarise the characteristics and risk of bias among contributing studies. | Figures 3 & 4 |
|  | 20b | Present results of all statistical syntheses conducted. If meta-analysis was done, present for each the summary estimate and its precision (e.g. confidence/credible interval) and measures of statistical heterogeneity. If comparing groups, describe the direction of the effect. | Figures 2, 3, & 5 |
|  | 20c | Present results of all investigations of possible causes of heterogeneity among study results. | Pages 15-16 & Table 1 |
|  | 20d | Present results of all sensitivity analyses conducted to assess the robustness of the synthesized results. | Figures 3 & 4 & Table 1 |
| Reporting biases | 21 | Present assessments of risk of bias due to missing results (arising from reporting biases) for each synthesis assessed. | Figures 3 & 4 & Table 1 |
| Certainty of evidence | 22 | Present assessments of certainty (or confidence) in the body of evidence for each outcome assessed. | Pages 15-16 |
| **DISCUSSION** | | |  |
| Discussion | 23a | Provide a general interpretation of the results in the context of other evidence. | Pages 24-28 |
|  | 23b | Discuss any limitations of the evidence included in the review. | Page 29 |
|  | 23c | Discuss any limitations of the review processes used. | Page 29 |
|  | 23d | Discuss implications of the results for practice, policy, and future research. | Page 29-30 |
| **OTHER INFORMATION** | | |  |
| Registration and protocol | 24a | Provide registration information for the review, including register name and registration number, or state that the review was not registered. | Page 7 |
|  | 24b | Indicate where the review protocol can be accessed, or state that a protocol was not prepared. | Page 7 |
|  | 24c | Describe and explain any amendments to information provided at registration or in the protocol. | Pages 11-12 |
| Support | 25 | Describe sources of financial or non-financial support for the review, and the role of the funders or sponsors in the review. | Page 31 |
| Competing interests | 26 | Declare any competing interests of review authors. | Page 31 |
| Availability of data, code and other materials | 27 | Report which of the following are publicly available and where they can be found: template data collection forms; data extracted from included studies; data used for all analyses; analytic code; any other materials used in the review. |  |

**Supplementary Table 3.** Characteristics of the included studies. Morris water maze (MWM), novel object recognition (NOR), Open field (OF), Radial arm maze (RAM), wild type (WT), Male (M), Female (F).

| **Study** | **Behavioural**  **tests** | **Mouse**  **strain** | **Sex** | **Age,**  **weight** | **Sample size** | **Microcoil size (μm)** | **Timepoint assessed** | **Quality score** |
| --- | --- | --- | --- | --- | --- | --- | --- | --- |
| [Andika, 2021. JNutr. 151: 722](https://doi.org/10.1093/jn/nxaa384) | NOR, Y maze | C57BL/6J | M | 4wks | 8-11 | 180 | 15 & 30d | 5 |
| [Ben-Ari, 2019. BrainRes. 1711: 193](https://doi.org/10.1016/j.brainres.2019.01.017) | NOR, OF, RAM | C57BL/Rcc | M | 3m | 15-16 | 180 | 28d | 4 |
| [Boehm-Sturm, 2017. Stroke. 48: 468](https://doi.org/10.1161/STROKEAHA.116.014394) | MWM, NOR | C57BL/6J | M | 8wks | 9-11 | 160 | ~28d | 5 |
| [Chen, 2016. JNeuroimmun. 299: 164](http://dx.doi.org/10.1016/j.jneuroim.2016.09.008) | MWM | C57BL/6J | M | 12wks, 30-35g | 10 | 180 | 41d | 5 |
| [Chen, 2017. SciRep. 7: 7758](https://www.nature.com/articles/s41598-017-08227-z) | RAM | C57BL/6J | M | 12 weeks, 30-35g | 10 | 180 | 35d | 4 |
| [Coltman, 2011. NeurobiolAge. 32: 2324e7](https://doi.org/10.1016/j.neurobiolaging.2010.09.005) | MWM, RAM | C57BL/6J | M | 3-4m, 25-30g | 12-24 | 180 | 30d | 5 |
| [Dam, 2017. BrainRes. 321: 201](https://doi.org/10.1016/j.bbr.2016.12.041) | MWM, Y maze | Mthfr^-/-^, Mthfr^+/+^ | M&F | 4wks | 12-13 | 180 | 28-36d | 4 |
| [Dong, 2011. Hyperten. 58: 635](https://doi.org/10.1161/HYPERTENSIONAHA.111.173534) | Y maze | C57BL/6J | M | 11wks | 15 | 160 | 14 & 21d | 3 |
| [Eguchi, 2018. BrainStim. 11: 959](https://doi.org/10.1016/j.brs.2018.05.012) | NOR, Y maze | C57BL/6 | M | 10-12wks | 12-17 | 180 | 28d | 4 |
| [Feng, 2021. FrontAgeNeuro. 13: 632374](https://doi.org/10.3389/fnagi.2021.632374) | RAM | C57BL/6J | M | 9-12wks, 25-30g | 11 | 180 | 28d | 6 |
| [Fuchtemeier, 2015. JCBFM. 35: 476](https://journals.sagepub.com/doi/10.1038/jcbfm.2014.221) | MWM | C57BL/6 | M | 10wks | 14-21 | 180 | 28d | 4 |
| [Han, 2019. CNSNeurosciTher. 25: 1042](https://doi.org/10.1111/cns.13189) | MWM, NOR, Y maze | C57BL/6J | M | 8-10wks, 25-28g | 10 | 180 | 60d | 5 |
| [Han, 2020. Theranost. 10: 2832](https://www.thno.org/v10p2832.htm) | MWM | PGC-α^f/f^ Eno2-Cre, WT | M | 12wks | 6-8 | 180 | 33d | 4 |
| [Hase, 2017. JNeuroinflamm. 14: 81](https://doi.org/10.1186/s12974-017-0850-5) | RAM | C57BL/6J | M | 9wks, 23-25g | 11-13 | 180 | 111d | 5 |
| [Hase, 2018. JCBFM. 38: 151](https://doi.org/10.1177/0271678X17694904) | RAM | C57BL/6J | M | 9ks, 23-25g | 11-13 | 180 | 111d | 5 |
| [Hase, 2019. NeuropAppNeurobio. 45: 681](https://onlinelibrary.wiley.com/doi/10.1111/nan.12550) | RAM | C57BL/6J | M | Adult | 10-13 | 180 | 111d | 5 |
| [Hattori, 2014. Stroke: 45: 3403](https://doi.org/10.1161/STROKEAHA.114.006265) | RAM | Sirt1-Tg, WT | M | Not provided | 15-17 | 180 | 37d | 3 |
| [Higaki, 2018. PLOSONE. 13: e0191708](https://doi.org/10.1371/journal.pone.0191708) | MWM | C57BL/6 | M | 10wks | 55-69 | 180 | 47d | 3 |
| [Higaki, 2018a. JAMA. 7: e008121](https://doi.org/10.1161/JAHA.117.008121) | MWM, Y maze | C57BL/6 | M | 10wks | 14-22 | 180 | 42d | 3 |
| [Holland, 2015. JCBFM. 35: 1005](https://doi.org/10.1038/jcbfm.2015.12) | MWM, RAM | C57BL/6J | M | 25-30g | 14 | 180 | 187d | 5 |
| [Iwanami, 2015. J Am Soc Hypert. 9: 250](http://dx.doi.org/10.1016/j.jash.2015.01.010) | MWM | C57BL/6 | M | 10wks, 23-25g | 6 | 180 | 42d | 3 |
| [Jadavji, 2015. BehavBrainRes. 283: 215](https://doi.org/10.1016/j.bbr.2015.01.040) | MWM, OF | Ung^-/-^, Ung^+/+^ | M&F | 4wks | 8 | 180 | 21-28d | 5 |
| [Khan, 2015. TransStrokeRes. 6: 69](https://doi.org/10.1007/s12975-014-0374-6) | NOR | C57BL/6J | M | 9-11wks | 5-7 | 180 | 28d | 6 |
| [Khan, 2018. TransStrokeRes. 9: 51](https://doi.org/10.1007/s12975-017-0555-1) | NOR | C57BL/6J | M | 10wks | 10 | 180 | 120d | 5 |
| [Kitamura, 2017. SciRep. 7: 4299](https://doi.org/10.1038/s41598-017-04082-0) | RAM | C57BL/6J | M | 4-5m, 25-30g | 10-12 | 180 | 90d | 5 |
| [Koizumi, 2018. NatComm. 9: 3816](https://www.nature.com/articles/s41467-018-06301-2) | NOR, Y maze | ApoE3-TR, APO4-TR, C57BL/6 | M | 3-4m | 10 | 180 | 28d | 5 |
| [Lee, 2019. JCBFM. 39: 44](https://doi.org/10.1177/0271678x17736963) | NOR, OF, Y maze | ApoE^-/-^, C57BL/6J | M | 10-12wks | 6-12 | 180 | 28d | 6 |
| [Maki, 2011. Stroke. 42: 1122](https://doi.org/10.1161/STROKEAHA.110.603399) | Y maze | AM-Tg, WT, C57BL/6J | M | 10-12wks, 22-29g | 17-26 | 180 | 28d | 3 |
| [Miki, 2009. JNeurosciRes. 87: 1270](https://doi.org/10.1002/jnr.21925) | MWM, OF | C57BL/6 | M | 16-18wks, 27-32g | 6-10 | 160 & 180 | 28-35d | 3 |
| [Miyamoto, 2013. Stroke. 44: 3516](https://doi.org/10.1161/STROKEAHA.113.002813) | Y maze | C57BL/6 | M | 10wks | 10 | 180 | 28d | 6 |
| [Miyanoharah, 2018. JNeurosci. 38: 3520](https://doi.org/10.1523/JNEUROSCI.2451-17.2018) | NOR, Y maze | TRPM2^-/-^, C57BL/6J | M | 9-12wks, 20-30g | 13-25 | 180 | 28d | 4 |
| [Mogi, 2018. Hypertens Res. 41: 809](https://doi.org/10.1038/s41440-018-0080-y) | MWM | C57BL/6 | M | 10wks, 23-25g | 23 | 180 | 42d | 4 |
| [Nishio, 2010. Stroke. 41: 1278](https://doi.org/10.1161/STROKEAHA.110.581686) | Barnes, RAM | C57BL/6J | M | 16wks, 25-35g | 14-15 | 180 | 6-10m | 2 |
| [Ohtomo, 2020. Trans Stroke Res. 11: 496](https://doi.org/10.1007/s12975-019-00734-7) | MWM, NOR, Y maze | C57BL/6J | M | 9wks | 12 | 180 | 56d | 5 |
| [Park, 2019. EurRevMedPharmSci. 23: 2587](https://doi.org/10.26355/eurrev_201903_17408) | MWM | C57BL/6 | M | 8wks | 7-9 | 180 | 33d | 4 |
| [Park, 2019a. BehavBrainRes. 365: 133](https://doi.org/10.1016/j.bbr.2019.03.013) | MWM | C57BL/6N | M | 8wks | 10 | 180 | 25-33d | 4 |
| [Patel, 2017. NeurolRes. 10: 910](https://doi.org/10.1080/01616412.2017.1355423) | NOR, RAM | C57BL/6J | M | 9-11wks, 24-29g | 6 | 180 | 39-40d | 4 |
| [Poh, 2021. MolPsych. 26: 4544](https://doi.org/10.1038/s41380-020-00971-5) | MWM, OF | AIM2^-/-^, C57BL/6 | M | 14-16wks, 24-30g | 11 | 180 | 26-30d | 3 |
| [Qin, 2017. Stroke. 48: 3336](https://doi.org/10.1161/strokeaha.117.018505) | RAM | C57BL/6 | M | 12-14wks, 23-28g | 8-9 | 180 | 35-37d | 6 |
| [Saggu, 2016. ActaNeuropath. 4: 76](https://doi.org/10.1186/s40478-016-0350-3) | OF, Y maze | GFAP-IkBα-dn, WT | M | 10-14wks | 12 | 170 | 42d | 5 |
| [Shibata, 2007. Stroke. 38: 2826](https://doi.org/10.1161/strokeaha.107.490151) | OF, RAM | C57BL/6 | M | 10-12ks, 24-29g | 10-13 | 180 | 30-40d | 3 |
| [Sigfridsson, 2018. SciRep. 8: 12552](https://doi.org/10.1038/s41598-018-30675-4) | RAM | GFAP-Nrf2, WT | M | 4-5m | 5-10 | 180 | 44d | 5 |
| [Sigfridsson, 2020. JNeuroinflam. 17: 367](https://doi.org/10.1186/s12974-020-02038-2) | RAM | Nrf2^-/-^, Nrf2^+/+^, WT | M | 6m, 25-44g | 7 | 180 | 44d | 5 |
| [Song, 2018. FrontPhysiol. 9: 662](https://doi.org/10.3389/fphys.2018.00662) | MWM, RAM | Tie2-GFP | M | 8-10wks, 25-28g | 6 | 180 | 47-49d | 4 |
| [Suzuki, 2021. JNeuroinflam. 18: 86](https://doi.org/10.1186/s12974-021-02135-w) | MWM, OF | Btg2^-/-^, WT | M | 9-11wks | 17-19 | 180 | 30-45d | 3 |
| [Toyama, 2014. ArtThromVascBiol. 34: 616](https://doi.org/10.1161/atvbaha.113.302440) | MWM, NOR, Y maze | ASK1^-/-^, C57BL/6J | M | 9wks | 10 | 160 | 14-21d | 2 |
| [Toyama, 2018. ArtThromVascBiol. 38: 1392](https://doi.org/10.1161/atvbaha.118.310822) | NOR, OF, Y maze | C57BL/6 | M | 9 weeks, 24-26g | 5-12 | 160 | 14-21d | 3 |
| [Tsai, 2015. JHyperten. 33: 1001](https://doi.org/10.1097/hjh.0000000000000529) | NOR | C57BL/6 | M | 12wks, 25-30g | 10 | 180 | 85d | 4 |
| [Tsai, 2017. Oncotarg. 8: 74320](https://doi.org/10.18632/oncotarget.20382) | Y maze | C57BL/6J | M | 12wks, 25-30g | 10 | 160 & 180 | 28d | 4 |
| [Wang, 2017. CNSNeurosciTher. 23:818](https://doi.org/10.1111/cns.12726) | MWM | C57BL/6 | M | 10-12wks, 22-25g | 12 | 200 | 45d | 3 |
| [Wang, 2019. Theranostics. 9: 4474](https://doi.org/10.7150/thno.31942) | RAM | C57BL/6 | M | 10-12wks, 22-27g | 8 | 180 | 44d | 6 |
| [Washida, 2010. Stroke. 41: 1798](https://doi.org/10.1161/STROKEAHA.110.583948) | Y maze | C57BL/6J | M | 9 weeks, 24-29g | 20 | 180 | 30d | 3 |
| [Wolf, 2017. FrontAgeNeurosci. 9: 191](https://doi.org/10.3389/fnagi.2017.00191) | OF, RAM | C57BL/6JRccHsd | F | 3 & 21m | 5-10 | 180 | 28d | 5 |
| [Yu, 2018. JMolNeurosci. 64: 449](https://doi.org/10.1007/s12031-018-1043-0) | RAM | Not provided | M | 9-11wks, 24-29g | 6 | 180 | 35d | 4 |
| [Yu, 2020. JNeurochem. 152: 350](https://doi.org/10.1111/jnc.14925) | RAM | Hv1^-/-^, C57BL/6 | M | 9-11wks, 22-28g | 10 | 180 | 35d | 6 |
| [Zhang, 2017. EurJInflamm. 17: 1](https://doi.org/10.1177/2058739219834832) | MWM, OF | C57BL/6 | M | 12wks, 25-30g | 16 | 180 | 26-27d | 5 |

**Supplementary Table 4.** Summary of recommendations.

Note: All rodent behavioural tasks require motor learning and attention and would therefore involve the Perceptual Motor Function and Attention human cognitive domains.

* indicates the possibility to modify the test to assess different aspects of cognition

| **Behavioural**  **test** | **Effective** | **Evidence for publication bias** | **Evidence of influential studies** | **Assesses** | **Human cognitive domains** | **Recommend** |
| --- | --- | --- | --- | --- | --- | --- |
| RAM | Yes | High | High | Spatial learning and memory^1^, Spatial working memory^2^ | Learning and memory > Executive function | Yes |
| NOR | Yes | Low | Low | Recognition memory^3^, Spatial memory*^4^, Social and emotional responses*^5^ | Learning and memory, Social cognition and emotions* | Yes |
| Y maze | Yes | High | Low | Spatial learning and memory^6^, Spatial working memory^7^, Spatial reference memory*^8^ | Learning and memory > Executive function | Yes |
| MWM | Yes | High | Low | Spatial learning and memory^9^, Spatial working memory^9,10^, Reversal learning*^10,11^ | Learning and memory > Executive function | Yes |
| MWM probe | Yes | Low | Low | Spatial reference memory^12,13^, Reversal learning*^10^ | Learning and memory > Executive function | Yes |
|  |  |  |  |  |  |  |
| Open field | - | - | - | Anxiety, locomotion, exploration^14^ | Social cognition and emotions | - |
| Barnes maze | - | - | - | Spatial learning and memory^15^ | Learning and memory | - |

**References**

1 Olton DS, Samuelson RJ. Remembrance of places passed: Spatial memory in rats. *J Exp Psychol Anim Behav Process* 1976; **2**: 97–116.

2 Olton DS, Collison C, Werz MA. Spatial memory and radial arm maze performance of rats. *Learn Motiv* 1977; **8**: 289–314.

3 Ennaceur A, Delacour J. A new one-trial test for neurobiological studies of memory in rats. 1: Behavioral data. *Behavioural Brain Research* 1988; **31**: 47–59.

4 Dere E, Huston JP, De Souza Silva MA. Integrated memory for objects, places, and temporal order: Evidence for episodic-like memory in mice. *Neurobiol Learn Mem* 2005; **84**: 214–221.

5 Hornoiu I, Gigg J, Talmi D. Quantifying how much attention rodents allocate to motivationally-salient objects with a novel object preference test. *Behavioural brain research* 2020; **380**.

6 Spence KW, Lippitt R. An experimental test of the sign-gestalt theory of trial and error learning. *J Exp Psychol* 1946; **36**: 491–502.

7 Wenk GL, Stoehr JD, Mobley SL, Gurney J, Morris RJ. Age-related decrease in vulnerability to excitatory amino acids in the nucleus basalis. *Neurobiol Aging* 1996; **17**: 1–7.

8 Kraeuter AK, Guest PC, Sarnyai Z. The Y-Maze for Assessment of Spatial Working and Reference Memory in Mice. In: *Pre-Clinical Models: Techniques and Protocols, Methods in molecular biology*. Springer, 2019, pp 105–111.

9 Morris R. Developments of a water-maze procedure for studying spatial learning in the rat. *J Neurosci Methods* 1984; **11**: 47–60.

10 Vorhees C V., Williams MT. Morris water maze: procedures for assessing spatial and related forms of learning and memory. *Nat Protoc* 2006; **1**: 848–858.

11 McMonagle-Strucko K, Fanelli RJ. Enhanced acquisition of reversal training in a spatial learning task in rats treated with chronic nimodipine. *Pharmacol Biochem Behav* 1993; **44**: 827–835.

12 Morris R. Developments of a water-maze procedure for studying spatial learning in the rat. *J Neurosci Methods* 1984; **11**: 47–60.

13 Bannerman DM, Good MA, Butcher SP, Morris RGM. Distinct components of spatial learning revealed by prior training and NMDA receptor blockade. *Nature 1995 378:6553* 1995; **378**: 182–186.

14 Hall CS, Ballachey EL. A study of the rat’s behavior in a field: A contribution to method in comparative psychology. *University of California Publications in Psychology* 1932; **6**: 112.

15 Barnes CA. Memory deficits associated with senescence: a neurophysiological and behavioral study in the rat. *J Comp Physiol Psychol* 1979; **93**: 74–104.
